# Mechanisms of VOR Suppression in Brainstem Pathology: Insights from the Absence of Anti-Compensatory Saccades Despite Normal VOR Gain

**DOI:** 10.3390/audiolres15060154

**Published:** 2025-11-12

**Authors:** Marco Tramontano, Laura Casagrande Conti, Nicola Ferri, Leonardo Manzari

**Affiliations:** 1 Translational Rehabilitation Group, Department of Biomedical and Neuromotor Sciences (DIBINEM), Alma Mater Studiorum University of Bologna, 40138 Bologna, Italy; 2 Unit of Occupational Medicine, IRCCS Azienda Ospedaliero-Universitaria di Bologna, 40138 Bologna, Italy; 3 IRCCS Santa Lucia Foundation, 00179 Rome, Italy; laura.casagrandeconti@gmail.com; 4 MSA ENT Academy Center, 03043 Cassino, Italy; lmanzari1962@gmail.com

**Keywords:** vestibulo-ocular reflex, anti-compensatory saccades, neurological disorders, SHIMP, vHIT, central vestibular dysfunction

## Abstract

**Background/Objective**: The Suppression Head Impulse Paradigm (SHIMP) is a specialized variant of the Head Impulse Test (HIT), designed to evaluate the suppression of the angular Vestibulo-Ocular Reflex (aVOR) by central mechanisms. These mechanisms are primarily mediated by brainstem structures, including the vestibular nuclei, their projections to ocular motor nuclei, and modulatory inputs from the cerebellum. Damage to these areas can impair the generation of anti-compensatory saccades (ACs), even when the peripheral vestibular apparatus remains intact. The present study explores this phenomenon in a cohort of patients with neurological disorders known to potentially involve the brainstem, including multiple sclerosis, severe traumatic brain injury, stroke, and Parkinson’s disease. **Methods**: This cross-sectional study included 119 patients with multiple sclerosis (PwMS), severe traumatic brain injury (PwTBI), stroke (PwS), and Parkinson’s disease (PwPD). The video Head Impulse Test was performed to assess the aVOR gain across all semicircular canals using both the HIMP and SHIMP. The presence, absence, or delay of ACs was systematically recorded. **Results**: Among the 119 patients evaluated (238 semicircular canals), 24 (20%) demonstrated normal aVOR gain but failed to generate ACs during SHIMP. The absence of ACs was observed in seven PwMS, five with PwTBI, six with PwS, and six with PwPD. **Conclusions**: The absence of ACs despite normal aVOR gain suggests a potential impairment in the central pathways controlling saccadic responses, independently of peripheral vestibular function. These findings underscore the clinical relevance of integrating the SHIMP into vestibular assessments to improve the identification of central vestibular dysfunction in neurological disorders.

## 1. Introduction

The Suppression Head Impulse Paradigm (SHIMP) is a variant of the Head Impulse Test (HIT) designed to evaluate the functionality of the angular Vestibulo-Ocular Reflex (aVOR) in a more nuanced manner [1]. VOR is crucial for maintaining visual stability during rapid head movements by generating compensatory eye movements in the opposite direction of head motion [2]. Traditional HIT focuses on detecting overt saccades when VOR fails [3]; however, it introduces a novel approach by requiring patients to fixate on a moving target during head impulses, eliciting anti-compensatory saccades in people with normal aVOR [4].

In recent years, evidence has supported the presence of semi-circular canal dysfunction in people with neurological disorders. Recent studies have shown that people with stroke (PwS) [5], multiple sclerosis (PwMS) [6], severe traumatic brain injury (PwTBI) [7], and Parkinson’s disease (PwPD) [8] can present aVOR alterations during the Head Impulse paradigm (HIMP) and SHIMP.

In patients with unilateral or bilateral vestibular hypofunction, the expected anti-compensatory saccades (ACs) may be absent or delayed despite a normal aVOR [9,10]. This phenomenon suggests a disruption of the central pathways responsible for the initiation of saccades [11].

Understanding the implications of absent ACs during SHIMP could significantly enhance diagnostic accuracy and clinical assessment, more than VOR gain, particularly in patients with central vestibular lesions [12]. The ability to differentiate between peripheral and central vestibular dysfunction based on video Head Impulse Test (vHIT) results can be particularly valuable in managing these populations [13]. Previous studies evaluating VOR gain in individuals with neurological disorders have primarily focused on quantitative assessment. While some studies [11,12,14] have analyzed compensatory saccades, none have specifically described the absence of ACs in the presence of physiological VOR gain. We hypothesized that this phenomenon may be present in patients with neurological disorders, suggesting a potential dysfunction in the central pathways responsible for saccadic initiation.

This study aimed to investigate whether the absence of ACs during the SHIMP occurs in patients with neurological disorders despite preserved VOR gain. By identifying this phenomenon, we sought to enhance our understanding of central vestibular dysfunction and improve the diagnostic accuracy of vestibular assessment in these populations.

## 2. Methods

This cross-sectional study received approval from the Local Independent Ethics Committee under protocol number Prot. CE/2022_011. All procedures adhered to ethical standards outlined by national and institutional guidelines on human experimentation, the World Medical Association Declaration of Helsinki, and the Strengthening the Reporting of Observational Studies in Epidemiology (STROBE) guidelines [15]. Written informed consent was obtained from all participants for the publication of results derived from their clinical examinations and instrumental tests.

### 2.1. Participants

Patients with multiple sclerosis (MS), severe traumatic brain injury (sTBI), Parkinson’s disease (PD), and stroke were recruited based on consecutive sampling at the hospital between March 2022 and July 2023.

Participants diagnosed with MS according to the McDonald criteria [16] were included if they presented a diagnosis of relapsing–remitting or secondary-progressive MS confirmed by a certified neurologist, an age of 18 years or older, and an Expanded Disability Status Scale score between 1 and 6 [17]. Exclusion criteria included the presence of psychiatric or neurological disorders other than MS, other pathological conditions and/or clinical disorders severe enough to interfere with cognitive functioning or the performance of motor or cognitive tasks, the occurrence of a clinical relapse in the three months prior to enrollment, steroid therapy administered in the 30 days preceding enrollment, a lower extremity fracture within three months prior to enrollment, or other medical conditions that could interfere with study procedures. A history of vestibular disorders was also considered an exclusion criterion.

Participants with sTBI were included if they were between 18 and 65 years old, had a Glasgow Coma Scale score of ≤8 at the time of injury, had a Level of Cognitive Functioning of 7 or higher [18], presented with disturbances in static and dynamic balance, and were able to understand verbal commands. Participants were also required to be able to walk without continuous physical assistance, with a Functional Ambulation Classification score greater than 3.

Participants with PD were included if they had no dementia, as indicated by a Mini-Mental State Examination score of greater than 25, had a Hoehn & Yahr stage of 2 or 3, and were able to walk without a device or without the need for continuous physical assistance (Functional Ambulation Classification > 3). Exclusion criteria included cognitive deficits affecting the ability to understand task instructions, severe vision impairment preventing the ability to focus on visual targets or impairing eye movements, history of ear surgery, chronic otitis media, deafness, vertigo, limited neck movement due to a neck injury, or the presence of neurological, orthopedic, or cardiac comorbidities.

Participants with first-ever stroke and unilateral hemiparesis were included if they were able to walk with or without a device and did not require continuous physical assistance, as indicated by a Functional Ambulation Classification score greater than 3 [19]. Exclusion criteria included cognitive deficits preventing task comprehension, defined as a Mini-Mental State Examination score lower than 24, severe unilateral spatial neglect, severe aphasia, and the presence of other neurological, orthopedic, or cardiac comorbidities.

### 2.2. Assessment

The vHIT (ICS Impulse, Otometrics/Natus, Denmark) was used to evaluate the aVOR gain for movements stimulating the six semicircular canals, assessing both the HIMP and SHIMP. The assessment protocol was designed to minimize variability and ensure accurate and reliable data collection. Evaluations were conducted by expert clinicians specializing in the vestibular field.

During the HIMP paradigm, patients were instructed to fixate on a point on the wall 1 m in front of them. Approximately 14 rapid, horizontal head turns (head impulses) were applied unpredictably to each side, with specific parameters: eye and head velocities were recorded for each impulse, with a normal VOR gain range defined as 0.76–1.29 for vertical canals and 0.80–1.29 for horizontal canals [20]. Head movements for vertical canal testing were adjusted to align with the Right Anterior-Left Posterior and Left Anterior-Right Posterior planes [3].

In the SHIMP, patients followed a red dot generated by a laser attached to their head during head impulses. Under physiological conditions, anti-compensatory saccades in the opposite direction to the aVOR are expected after a short latency [9]. A normal VOR gain range in the SHIMP was defined as 0.66–1.29. VOR gain outside these ranges was classified as hypo-gain or hyper-gain based on direction [20].

### 2.3. Statistical Analysis

To evaluate the absence of ACs, each raw data entry was screened by two experts in the field for the presence or absence of saccades, ensuring accuracy in classification. Statistical analyses were conducted to evaluate differences in SHIMP VOR gain among the four neurological conditions and to assess correlations between SHIMP gain and the absence of ACs. A one-way analysis of variance (ANOVA) was performed to determine whether SHIMP horizontal left and right gain differed significantly among the groups. Post hoc pairwise comparisons were conducted using Tukey’s Honestly Significant Difference test to identify specific differences between conditions. Additionally, Pearson correlation analysis was used to assess the relationship between SHIMP horizontal gain and the absence of ACs.

## 3. Results

One-hundred and nineteen patients were evaluated in this study, encompassing 238 semicircular canals assessed using the SHIMP. Twenty-seven PwMS (mean age 47.93 ± 8.5), 22 PwTBI (mean age 42 ± 15.02), 36 with stroke (mean age 55.11 ± 15.09), and 35 PwPD (mean age 69.9 ± 8.4), clinical and demographic characteristics are reported in Table 1.

**Figure 1:**
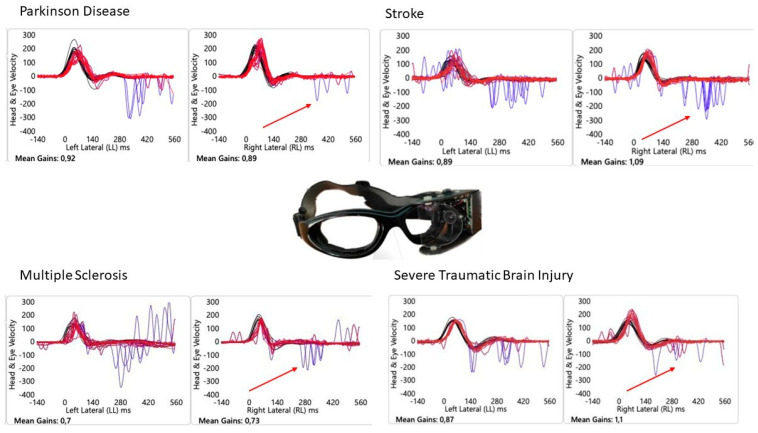
Suppressed Head Impulse Paradigm in People with Neurological disorders. Head velocity and eye velocity are represented by the red and blue lines, respectively. Red arrow indicates the lack of Anti-compensatory saccades in normal VOR gain during the SHIMP. A representative case for each disease.

The results revealed that 24 out of 119 patients (20%) demonstrated a normal aVOR gain in the SHIMP but did not exhibit any ACs. Among those with absent ACs, 7 were PwMS, 5 sTBI, 6 stroke and 6 PwPD (representative cases are reported in Figure 1). The ANOVA for the SHIMP horizontal left gain yielded a statistically significant result (*p* = 0.041), indicating differences among the groups. The ANOVA for the SHIMP VOR gain of the right horizontal semicircular canal showed no significant differences (*p* = 0.067). Post hoc analysis using Tukey’s test revealed that for SHIMP VOR gain on the left side, PwMS had a significantly higher gain than PwTBI (*p* < 0.05), and stroke patients had a significantly higher gain than PwTBI (*p* < 0.05). No significant differences were found between the PwPD group and the other groups. For SHIMP VOR gain on the right side, PwTBI had a significantly lower gain compared to PwMS (*p* < 0.05) and stroke patients (*p* < 0.05), whereas no significant differences were found for PD compared to other groups. Moderate correlations were found between the SHIMP VOR gain on either side and the absence of ACs (right side: r = −0.42, *p* = 0.012; left side: r = −0.36, *p* = 0.038).

## 4. Discussion

This study identified the absence of ACs in individuals with neurological disorders who demonstrated normal aVOR gain during the SHIMP. The findings revealed that 20% of the evaluated patients (24/119) did not exhibit ACs despite a preserved aVOR gain. This phenomenon has been observed across different neurological conditions, including MS, sTBI, stroke, and PD. These results align with previous evidence indicating that saccadic abnormalities can occur in neurological disorders even when the vestibular reflex is functionally intact [21], which is consistent with the modest correlation we found between the aVOR gain and the ACs in our population. In particular, the absence of ACs suggests a possible disruption of the central pathways responsible for saccadic generation, which may not be directly reflected in VOR gain measurements alone. The SHIMP was designed as an alternative to the HIMP to provide additional information about saccadic function in individuals with preserved vestibular function. Under normal conditions, aVOR suppression should lead to the generation of ACs, but in the present study, a significant proportion of patients with neurological disorders failed to exhibit these responses. Hawkins and colleagues [14] showed that individuals with PD exhibited reduced peak AC velocities and prolonged latencies compared to healthy controls, despite no significant difference in VOR gain. The absence or delay of ACs in the SHIMP has potential implications for identifying central vestibular dysfunction. Unlike peripheral vestibular loss, where compensatory saccades are expected due to VOR impairment, the current findings suggest that certain neurological disorders disrupt saccadic initiation even when the VOR is intact. This is consistent with previous studies showing increased saccadic variability and prolonged latencies in central vestibular disorders [12].

Saccadic impairments, including increased saccadic latencies and altered compensatory saccade characteristics were reported in PwMS [22]. These impairments are often related to lesions in the cerebellar peduncles, leading to saccadic dysmetria. Demyelination and neurodegeneration in MS may disrupt the neural circuits responsible for saccade initiation, contributing to the absence of ACs despite preserved aVOR function. PwTBI can exhibit delayed saccadic responses, possibly due to diffuse axonal damage that involves oculomotor pathways and the control of saccadic generation. Research indicates that individuals with mild TBI show impairments in eye movements, including increased saccadic latencies and decreased accuracy, which can interfere with tasks requiring precise visual attention [23].

In Stroke, there can be impairments in all critical regions for generating saccades. Such damage may lead to inconsistent saccadic execution, explaining the absence of ACs despite a preserved VOR, indeed impairment of saccadic eye movements is associated with the risk of developing cerebral infarction due to delayed cerebral ischemia [24].

The absence of ACs in individuals with neurological disorders despite normal aVOR gain underscores the importance of incorporating saccadic response assessments into vestibular testing. While traditional vHIT interpretation relies primarily on VOR gain, evaluating ACs may provide additional insights into central vestibular dysfunction.

These findings are particularly relevant for differentiating between peripheral and central vestibular dysfunctions. Unlike patients with unilateral vestibular hypofunction, who exhibit compensatory saccades to correct for gaze instability, patients with central lesions may fail to generate appropriate ACs despite intact VOR function. This distinction supports the clinical utility of the SHIMP in assessing oculomotor function beyond VOR gain alone.

However, some limitations must be acknowledged. First, the cross-sectional study design does not allow us to monitor the evolution of saccadic response in the SHIMP assessment or to attribute prognostic value to our findings. Second, the sample size is relatively small, which increases the risk of not capturing all the characteristics present in the population. Lastly, we were unable to perform a multivariate analysis to identify potential confounders (e.g., age, sex, disease duration) due to the limited sample size. Finally, while our findings support brainstem-related dysfunction, alternative explanations such as cerebellar involvement, fatigue, or attentional deficits should also be considered.

## 5. Conclusions

This study identifies a previously underexplored phenomenon: the absence of ACs in neurological disorders despite preserved VOR gain during SHIMP testing; this may reflect dysfunction of brainstem pathways involved in saccadic initiation. These findings suggest that central vestibular impairment can manifest independently of peripheral VOR deficits, highlighting the potential of SHIMP as a non-invasive diagnostic tool for detecting brainstem pathology. Given the brainstem’s critical role in integrating vestibular, visual, and proprioceptive inputs for gaze and postural control, future research should examine SHIMP parameters as biomarkers for a range of brainstem-mediated disorders impacting hearing, balance, speech, and swallowing.

## Figures and Tables

**Table 1:** Demographic and clinical characteristics of participants across neurological conditions.

	Multiple Sclerosis	Severe Traumatic Brain Injury	Stroke	Parkinson’s Disease
Sample Size (n)	27	22	36	35
Age (years, mean ± SD)	47.93 ± 8.51	42 ± 15.02	55.11 ± 15.09	69.9 ± 8.4
Sex (Female, %)	66.67%	14.3%	30.5%	31.4%
HIMP aVOR Gain (mean ± SD)				
Left Anterior	0.78 ± 0.22	0.81 ± 0.15	0.81 ± 0.20	0.79 ± 0.19
Right Anterior	0.86 ± 0.14	0.71 ± 0.18	0.83 ± 0.26	0.85 ± 0.25
Horizontal Left	0.92 ± 0.19	0.87 ± 0.15	0.9 ± 0.12	0.94 ± 0.16
Horizontal Right	0.98 ± 0.24	0.97 ± 0.21	0.98 ± 0.14	0.99 ± 0.20
Left Posterior	0.82 ± 0.12	0.86 ± 0.15	0.91 ± 0.16	0.85 ± 0.22
Right Posterior	0.76 ± 0.17	0.91 ± 0.25	0.79 ± 0.12	0.79 ± 0.19
SHIMP aVOR Gain (mean ± SD)				
Horizontal Left	0.78 ± 0.21	0.81 ± 0.26	0.88 ± 0.20	0.85 ± 0.19
Horizontal Right	0.87 ± 0.23	0.74 ± 0.23	0.77 ± 0.19	0.85 ± 0.2

## Data Availability

The original data presented in the study are included in the article, further inquiries can be directed to the corresponding authors.
